# Development of a framework and coding system for modifications and adaptations of evidence-based interventions

**DOI:** 10.1186/1748-5908-8-65

**Published:** 2013-06-10

**Authors:** Shannon Wiltsey Stirman, Christopher J Miller, Katherine Toder, Amber Calloway

**Affiliations:** 1 Women’s Health Sciences Division, National Center for PTSD, Boston, MA, USA; 2 VA Boston Healthcare System, Boston, MA, USA; 3 Department of Psychiatry, Boston University, Boston, MA, USA; 4 Department of Psychiatry, University of Pennsylvania, Philadelphia, PA, USA; 5 VA Center for Organization, Leadership and Management Research, Boston, MA, USA; 6 Department of Psychology, University of Massachusetts Boston, Boston, MA, USA

**Keywords:** Implementation, Modification, Adaptation, Sustainability

## Abstract

**Background:**

Evidence-based interventions are frequently modified or adapted during the implementation process. Changes may be made to protocols to meet the needs of the target population or address differences between the context in which the intervention was originally designed and the one into which it is implemented [*Addict Behav* 2011, **36**(6):630–635]. However, whether modification compromises or enhances the desired benefits of the intervention is not well understood. A challenge to understanding the impact of specific types of modifications is a lack of attention to characterizing the different types of changes that may occur. A system for classifying the types of modifications that are made when interventions and programs are implemented can facilitate efforts to understand the nature of modifications that are made in particular contexts as well as the impact of these modifications on outcomes of interest.

**Methods:**

We developed a system for classifying modifications made to interventions and programs across a variety of fields and settings. We then coded 258 modifications identified in 32 published articles that described interventions implemented in routine care or community settings.

**Results:**

We identified modifications made to the content of interventions, as well as to the context in which interventions are delivered. We identified 12 different types of content modifications, and our coding scheme also included ratings for the level at which these modifications were made (ranging from the individual patient level up to a hospital network or community). We identified five types of contextual modifications (changes to the format, setting, or patient population that do not in and of themselves alter the actual content of the intervention). We also developed codes to indicate who made the modifications and identified a smaller subset of modifications made to the ways that training or evaluations occur when evidence-based interventions are implemented. Rater agreement analyses indicated that the coding scheme can be used to reliably classify modifications described in research articles without overly burdensome training.

**Conclusions:**

This coding system can complement research on fidelity and may advance research with the goal of understanding the impact of modifications made when evidence-based interventions are implemented. Such findings can further inform efforts to implement such interventions while preserving desired levels of program or intervention effectiveness.

## Background

Evidence-based programs and interventions are frequently modified during the implementation process to address differences between the context in which the intervention was originally designed and tested, and the one into which it is ultimately implemented [1]. Descriptions of modifications in the research literature range broadly from slight changes in terminology or delivery in different languages, to removal of core components or integration with other interventions. Modifications can include adaptations, which are planned or purposeful changes to the design or delivery of an intervention, but they can also include unintentional deviations from the interventions as originally designed. That is, some modifications occur with the intention to retain fidelity to the fundamental elements or spirit of the intervention, whereas others may be unplanned changes made in reaction to a specific circumstance. Some may be relatively minor, while others might represent a significant change. Such variation in the nature of modifications can have very different implications for outcomes of interest. While some modifications might facilitate implementation and sustainability by improving the fit between the intervention and the target population or the context into which it is introduced, modifications may also erode treatment integrity. However, little research has been conducted to determine whether modifications of different natures compromise or enhance the desired benefits of interventions. Assessing fidelity, which comprises adherence to the intervention components, competence or skill with which the intervention is delivered, and differentiation from other treatments [2], by itself may fail to capture certain types of modifications (*e.g*., minor changes to terminology or language). While the recommended considerations for fidelity include unique and essential elements, necessary but not unique, acceptable but not necessary, and proscribed elements [3], most fidelity instruments do not contain an exhaustive listing of acceptable and proscribed behaviors. Thus, fidelity monitoring alone will not facilitate an understanding of whether different types of modifications are detrimental, non-detrimental or enhancements [4,5]. Without a better understanding of the nature and impact of modification, and the levels of fidelity necessary to promote desired outcomes, it is difficult to determine the best course of action with respect to the implementation of complex interventions in different contexts. This manuscript presents a comprehensive framework and model for classifying a broad range of modifications that may be made to evidence-based interventions. Such a framework, by quantifying the specific types and levels of modifications, can allow for more precise determination of the effects of such modifications on clinical or implementation outcomes of interest.

While the case has been made both for strict fidelity to interventions and for modifying interventions as necessary [4,6-10], few studies have examined the impact of modifications to treatments on health-related behaviors or outcomes. Among those studies, results have not been consistent. Levitt and colleagues compared outcomes of an intervention for post-traumatic stress disorder (PTSD) that included the option to use certain prescribed modifications, such as repeating or skipping modules, with clinical outcomes from a randomized controlled trial [11]. In this study, levels of fidelity to core intervention components remained high when the intervention was delivered with modifications, and PTSD symptom outcomes were comparable to those in a controlled clinical trial [11]. Galovski and colleagues also found positive outcomes when a highly specified set of adaptations were used in a different PTSD treatment [12]. Other studies have demonstrated similar or improved outcomes after modifications were made to fit the needs of the local audience and expand the target population beyond the original intervention. For example, an enhanced outcome was demonstrated after modifying a brief HIV risk-reduction video intervention to match presenter and participant ethnicity and sex [13]; effectiveness was also retained after modifying an HIV risk-reduction intervention to meet the needs of five different communities [14]. However, in other studies, modifications to enhance local acceptance appeared to compromise effectiveness. For example, Stanton and colleagues modified a sexual risk reduction intervention that had originally been designed for urban populations to address the preferences and needs of a more rural population, but found that the modified intervention was less effective than the original, unmodified version [15]. Similarly, in another study, cultural modifications that reduced dosage or eliminated core components of the Strengthening Families Program increased retention but reduced positive outcomes [16].

A challenge to a more complete understanding of the impact of specific types of modifications is a lack of attention to their classification. Some descriptions of intervention modifications and adaptations have been published (c.f. [17-19]), but there have been relatively few efforts to systematically categorize them. Researchers identified modifications made to evidence-based interventions such as substance use disorder treatments [1] and prevention programs [20] through interviews with facilitators in different settings. Others have described the process of adaptation (e.g., [21,22]). For example, Devieux and colleagues [23] described a process of operationalizing the adaptation process based on Bauman and colleagues’ framework for adaptation [8], which includes efforts to retain the integrity of an intervention’s causal/conceptual model. Other researchers [24-26] have also made recommendations regarding specific processes for adapting mental health interventions to address individual or population-level needs while preserving fidelity. Some work has been done to characterize and examine the impact of modifications made at the individual and population level. For example, Castro, Barrera and Martinez presented a program adaptation framework that described two basic forms of cultural adaptation: the modification of program content and modification of program delivery, and made distinctions between tailored and individualized interventions [27]. A description of person-centered interventions similarly differentiates between tailored, personalized, targeted and individualized interventions, all of which may actually lie on a continuum in terms of their complexity and comprehensiveness [27].

While these existing recommendations and models for adaptation provide critical guidance regarding the process of adaptation, particularly to improve the cultural relevance and individual-level ‘fit’ of interventions, adaptations may also occur to address provider needs or constraints in the intervention or program setting or healthcare system. Relatively little research has been conducted to empirically classify the nature of the full range of modifications made to interventions in routine care settings. Recognizing the benefits to identifying common types of modifications to evidence-based interventions, Hill and colleagues identified the types of modifications made for a single evidence-based prevention program in a statewide implementation [20]. Although they suggested that their study was a starting point in the development of a set of modification categories that generalize across interventions, the modifications that they identified were highly specific to the intervention that they examined. A more general and developed taxonomy can facilitate efforts to understand the nature of modifications that are made, whether by design or happenstance, as well as the impact of different types of modifications on implementation and health-related outcomes of interest. As a next step toward this understanding, it is necessary to identify the types of modifications that may occur when implementing interventions under a broad set of circumstances and in a variety of settings.

The purpose of this study was to develop a coding scheme to characterize modifications made to evidence-based interventions when they are implemented in contexts or with populations that differ from that in which they were originally developed or tested. Such a system can facilitate more systematic study of the types of modifications that are most commonly made across different contexts, populations and interventions. Additionally, it can provide a way to study the impact of different types of modifications on outcomes of interest. Hill and colleagues described a theory that a few types of modifications are likely to comprise the majority of all modifications that occur in practice [20]. Identifying what those modifications are, and what their impacts are for different interventions, can assist intervention developers and those who implement the interventions in determining and facilitating the range of modifications that are acceptable—and in preventing those that are not. We therefore sought to identify examples of a variety of modifications that practitioners, treatment developers, and other stakeholders made to a diverse set of interventions and programs. We intended this framework to apply in particular to three types of programs and interventions outlined by Scheirer [28]: those implemented by individual providers; programs requiring coordination among multiple staff; and new procedures aimed at targeting individual behaviors or behavioral health conditions.

## Method

**Figure 1:**
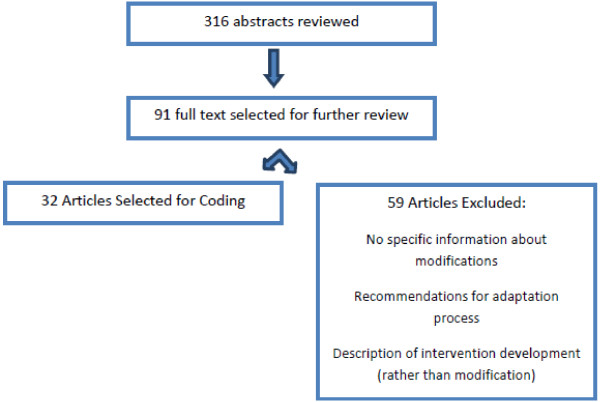
Article selection procedure and results.

In developing the coding system, we searched the literature for articles published or in press before June, 2012, that assessed or described modifications to interventions implemented in routine service settings. We searched the following databases: Medline, ISI, PsycInfo, Academic Search Premier, Health Source, ERIC, Pubmed and Google Scholar, using the terms ‘modif’ or ‘adapt’ and ‘evidence based treatment’ or ‘evidence-based intervention.’ We also employed a snowballing strategy, in which we searched the reference sections of articles that we identified as well as theoretical papers on implementation that discussed modification and adaptation. Two authors (SWS, AC) reviewed abstracts and full text articles when necessary to determine their eligibility for this project and discussed one difference of opinion regarding inclusion with the rest of the study team. We included articles that provided sufficient detail about one or more modifications to facilitate coding. Modifications could be either adaptations (intentional, planned changes that typically included an effort to preserve fidelity) or changes that were made without premeditation during the delivery of the intervention. Articles were excluded if they assessed fidelity but did not describe modifications; if the interventions were developed, as opposed to being modified, for the purpose of the study or implementation project; or if they only provided recommendations for adaptation of an intervention without describing at least one specific modification that was made during the course of a research or implementation effort (Figure 1).

Once identified, articles were examined and segmented into discrete units for coding. One team member (SWS) segmented the articles into separate descriptions of modifications, and the remaining team members reviewed the entire articles and provided feedback regarding the accuracy of the segmentation process. Due to the dearth of previous work that has been done to actually classify a broad range of modifications, we used an analytic approach that was rooted in grounded theory [29,30] to code the data from these articles. Using an iterative process, the study team examined the subset of identified segments that described modifications to identify emergent themes or categories. We then applied these themes to additional segments, allowing additional categories to emerge until theoretical saturation was achieved and a comprehensive coding scheme was developed. We specified characteristics of each category of modifications and ensured that the categories were mutually exclusive and exhaustive by combining or revising redundant codes. We also provided a draft of the framework and codebook to seven implementation researchers who were interested in the subject of modification and requested feedback. No additional codes or alternative classification structures were suggested, although some suggested combining some similar constructs into a single code and identified aspects of the codebook that could be clarified. After this feedback was incorporated, two team members (SWS, KT) then re-examined the article data, such that all segments identified in each article were coded with the finalized codebook. The two raters overlapped on 20% of the identified segments, and we computed Cohen’s kappa coefficients [31] to determine interrater reliability for each rating category.

## Results

A total of 32 articles were identified [9,11,13,15-18,20,32-51], which described 258 unique modifications (see Table 1). The types of intervention included preventive and health promotion interventions (n = 15), mental or behavioral health (n = 13), behavioral medicine (n = 3), and a multidimensional complex care coordination intervention (n = 1). Settings in which the interventions were delivered included hospitals and medical clinics, mental health clinics, substance abuse treatment programs, human service organizations, housing shelters, community organizations, employment settings, bars, and schools. Twenty-three articles provided author descriptions of modifications, four identified modifications through interviews with providers, two utilized observation or fidelity rating, and three based findings on a combination of observation and interviews.

**Table 1:** Articles included in coding procedure

**Citation**	**Type of program**	**Source of modification data**
Aarons et al., [35]	Mental health	Behavioral observation
Blasinsky, Goldman & Unutzer, [46]	Collaborative care/mental health	Observation and interview
Devieux et al., [23]	HIV/behavioral medicine	Author description
Dushay et al., [41]	HIV Prevention	Author description
Hasson, Blomberg & Duner, [44]	Complex care	Observation and interview
Hill, Maucione & Hood, [20]	Substance abuse prevention	Clinician interview
Hinton et al., [32]	Mental health	Author description
Holliday et al., [51]	Health promotion	Observation and interview
Kalichman et al., [13]	HIV Prevention	Author description
Kaysen et al., [17]	Mental health	Author description
Kelly et al., [9]	HIV Prevention	Author description
Kennedy et al., [14]	HIV Prevention	Author description
Kumpfer, Smith & Bellamy, [16]	Prevention	Author description
Leerlooijer et al., [19]	Sexual risk prevention	Author description
Levitt et al., [11]	Mental health	Author description
Lundgren et al., [1]	Substance abuse	Clinician interview
Lyon et al., [38]	Mental health	Author description
Malow et al., [39]	HIV Prevention	Author description
McCabe et al., [33]	Mental health	Author description
McIntyre, [38]	Mental health	Author description
Melde, Esbensen & Tusinski, [47]	Prevention	Behavioral observation
Miller, [42]	HIV Prevention	Author description
Nastasi et al., [21]	Sexual risk prevention	Author description
Noonan et al., [49]	Sexual violence prevention	Clinician interview
Owczarzak & Dickson-Gomez, [37]	HIV Prevention	Clinician interview
Remien et al., [48]	HIV/behavioral medicine	Author description
Salerno et al., [18]	Mental health	Author description
Stanton et al., [15]	Sexual risk prevention	Author description
Tortolero et al., [22]	Sexual risk prevention	Author description
Webster-Stratton & Herman, [50]	Mental health	Author description
Webster-Stratton & Reid, [34]	Mental health	Author description
Williams & Williams, [45]	Behavioral medicine	Author description

### Classification of modifications

**Figure 2:**
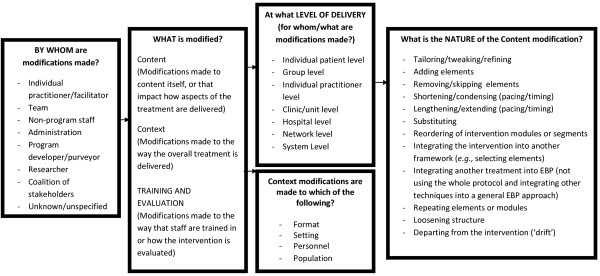
System of classifying modifications to evidence-based programs or interventions.

Our coding process resulted in the identification of modifications to the context of program or intervention delivery, modifications to the intervention or program content itself, and modifications made during an implementation effort to training or evaluation processes. Furthermore, we included a code specifying who made the decision to make each modification. Figure 2 represents the coding system that emerged from this process, which is described in greater detail below. A comprehensive coding manual that includes decision rules and instructions regarding how to code each level is available by request from the first author. Contextual modifications include format, setting, channel of delivery and intervention recipients, and are about ‘setting the stage’ for an intervention to be delivered. Content modifications focus on the actual delivery of the intervention content. Training and evaluation modifications represent changes made ‘behind the scenes’ during an implementation effort. Although modifications to context and training/evaluation codes were not always accompanied by substantial changes to the intervention content, we included them because it is possible that such changes could have an impact on fidelity, clinical outcomes, or the success of an implementation effort. Table 2 includes the frequency with which each modification occurred, along with rater agreement statistics.

**Table 2:** Modifications and adaptations made to programs and interventions

**Decision maker (overall kappa = 0.80, n = 261)**	**Frequency**	**% of total modifications**	
Individual practitioner^a^	55	21	
Team or group of practitioners	24	9	
Administrator	2	<1	
Researcher	22	8	
Intervention developer or purveyor^b^	70	27	
Coalition of stakeholders^c^	76	29	
Unknown/insufficient information	12	5	
**Contextual modifications (overall kappa = 1.00, n = 41)**	**Frequency**	**% of contextual modifications**	**% of total modifications**
Format	8	25	3
Setting	6	19	2
Personnel	10	3	4
Population	8	25	3
**Training and evaluation Processes**	**9**	**-**	**3**
**Content modifications (n = 217)**	**Frequency**	**% of content modifications**	**% of total modifications**
**Level (overall kappa = 0.79)**			
Individual recipient	14	6	5
Cohort	14	6	5
Population^d^	71	33	28
Provider/facilitator	20	9	8
Unit^e^	38	18	15
Hospital/organization	22	10	9
Network/community^f^	38	18	15
**Nature of modification (overall kappa = 0.87)**			
Tailoring/tweaking/refining^f^	73	34	28
Adding elements^g^	62	29	24
Removing elements^g^	33	15	13
Shortening/condensing^h^	17	8	7
Lengthening/extending	12	6	5
Substituting elements	5	2	2
Re-ordering elements	4	2	2
Integrating another approach into the intervention	3	1	1
Integrating the intervention into another approach	2	1	1
Departing from the intervention (“Drift”)	1	<1	<1
Loosening structure	1	<1	<1
Repeating elements	1	<1	<1

Note: Three modifications that were identified in the articles did not contain sufficient detail to be rated.

^a^Raw agreement for this category was 100%.

^b^Raw agreement for this category was 82%.

^c^Raw agreement for this category was 73%.

^d^Raw agreement for this category was 87%.

^e^Raw agreement for this category was 67%.

^f^Raw agreement for this category was 91%.

^g^Raw agreement for these categories was 83%.

^h^Raw agreement for this category was 86%.

### By whom was the decision to modify made?

This code indicates the individual or group of individuals who made the decision regarding whether or how to modify the intervention. Cohen’s kappa for this code was 0.80, indicating substantial agreement.

1. Provider, practitioner, or facilitator: The individual who delivers the intervention made the modification.

2. Team/multiple providers: A group of providers modified the treatment (*e.g*., either an intervention that requires multiple providers is modified by those providers, or a unit of providers decide together to deliver a program or intervention in a different way).

3. Administrator or supervisor: The individual responsible for oversight of an individual provider, team, unit, organization or system decided how to modify the intervention or program.

4. Researcher: A researcher determined how to modify a program or intervention for the purposes of research (*e.g*., to study the impact of a particular adaptation or set of adaptations).

5. Purveyor or intervention developer: The individual who developed the intervention or an (often external) individual with expertise in the intervention who was tasked with supporting the implementation determined how to adapt or modify the treatment. If the purveyor and researcher are the same individual, the coding decision is made based on whether the modification is for research or implementation purposes.

6. Coalition of Stakeholders: A group of stakeholders actively participated in the decision-making regarding the types of modifications that are made to an intervention. If the purveyor or researchers used focus groups, interviews, or other means of gathering input to guide their decisions regarding modifications, this code was NOT used, unless stakeholders also directly participated in the process of using that information to adapt the intervention.

### Contextual modifications

Similar to Castro and colleagues’ description of differing forms of delivery [27], contextual modifications were defined as changes made to delivery of the same program content, but with modifications to the format or channel, the setting or location in which the overall intervention is delivered, or the personnel who deliver the intervention. We also include in this category the population to which an intervention is delivered. Modifications were only coded as contextual if an intervention was specifically designed for a particular context or population and then applied elsewhere or delivered in a different format than originally designed. Modifications were considered to be contextual if one of the elements described below was changed, whether or not alterations to the content of the intervention were made. When content-level changes were also made, they were coded separately. A total of 41 contextual modifications (16% of the total sample of modifications) were described in the sample of articles. The subset of segments that was double-coded for reliability purposes indicated perfect agreement for the presence of contextual modifications.

1. Format: Changes are made to the format or channel of treatment delivery (*e.g*., a treatment originally designed to be used one-on-one that is now delivered in a group format).

2. Setting: The intervention is being delivered in a different setting or location (*e.g*., a treatment originally designed to be used in a mental health clinic setting that is now delivered in primary care).

3. Personnel: The intervention is being delivered by personnel with different characteristics (*e.g*., a treatment originally designed to be administered by a mental health professional is now delivered by clergy).

4. Population: An intervention that was specifically developed to target a particular population is being delivered to a different population than originally intended (*e.g*., an intervention developed for patients with Borderline Personality Disorder is now being delivered to individuals with Substance Dependence).

### Modifications to training and evaluation processes

Changes made to the procedures for training personnel or evaluating the program are classified separately from content or contextual modifications, as they occur ‘behind the scenes’ and do not necessarily impact intervention content or the context of delivery. Examples include expanding training from a single day to a three-day workshop, or making changes to the type of evaluation data or procedures for collecting evaluation data.

### Content modifications

Content modifications are changes made to the intervention procedures, materials or delivery. They appear to occur at multiple levels and in differing contexts, ranging from changes made for an individual recipient to changes made uniformly across an entire network, community or system. Therefore, we included a code for both the level at which the modification was made (*e.g*., for a single patient vs. across the entire clinic), and the nature of the modification itself. A total of 217 content modifications (84% of all identified modifications) were described in the articles that were reviewed. Table 2 summarizes the frequency with which modification occurred at the following levels. Cohen’s kappa for agreement on levels was 0.79, indicating substantial agreement [52].

### Levels at which content modifications occur

1. Individual recipient level: The intervention is modified for a particular recipient (*e.g*., simplifying language if a patient has cognitive impairment or if language barriers exist; changes to increase cultural relevance for an individual recipient).

2. Cohort level: The intervention is modified for individuals grouped within the intervention setting into a treatment group, a class, or other type of cohort (*e.g*., a specific psychotherapy group, grade or classroom).

3. Population level: The intervention is modified for application to a particular cultural, ethnic, clinical or social group (*e.g*., repetition of intervention components for all patients with cognitive impairments; development of culturally relevant vignettes to be used with all individuals of a particular ethnic identity).

4. Provider/facilitator level: Modifications are made by a clinician/facilitator for all of their participants (*e.g*., ‘I never set an agenda when I do cognitive therapy’).

5. Unit level: A modification is made by all of the facilitators in a unit (*e.g*., clinic/department/grade) within a larger organization (*e.g*., ‘We can only do 60-minute intervention sessions instead of 90-minute sessions in our clinic’).

6. Hospital/Organization level: Modifications are made by an entire organization.

7. Network/Community level: Modifications are applied by an entire network or system of hospitals/clinics/schools (*e.g*., a Veterans Affairs VISN; school district) or community.

### Types of content modifications

We identified 12 different types of content modifications. Cohen’s kappa for the nature of modifications was 0.87, suggesting that rater agreement for these categories was in the ‘almost perfect’ range [52]. Regarding reliability for individual codes, raw agreement was at least 80% for each code that was applied more than 15 times in our dataset; less frequently-applied codes were not subjected to reliability analyses.

1. Tailoring/tweaking/refining: This code was assigned to any minor change to the intervention that leaves all of the major intervention principles and techniques intact while making the intervention more appropriate, applicable or acceptable (*e.g*., modifying language, creating slightly different versions of handouts or homework assignments, cultural adaptations).

2. Adding elements (intervention modules or activities): Additional materials or activities are inserted that are consistent with the fundamentals of the intervention (*e.g*., adding role play exercises to a unit on assertiveness in a substance abuse prevention intervention).

3. Removing elements (removing/skipping intervention modules or components): Particular elements of the intervention are not included (*e.g*., leaving out a demonstration on condom use in an HIV prevention intervention for adolescents).

4. Shortening/condensing (pacing/timing): A shorter amount of time than prescribed is used to complete the intervention or intervention sessions (*e.g*., shorter spacing between sessions, or shortening sessions, offering fewer sessions, or going through particular modules or concepts more quickly without skipping material).

5. Lengthening/extending (pacing/timing): A longer amount of time than prescribed by the manual/protocol is spent to complete intervention or intervention sessions (*e.g*., greater spacing between sessions, longer sessions, more sessions, or spending more time on one or more modules/activities or concepts).

6. Substituting elements: A module or activity is replaced with something that is different in substance (*e.g*., replacing a module on condoms with one on abstinence in an HIV prevention program).

7. Re-ordering elements: Modules/activities or concepts are completed in a different order from what is recommended in the manual/protocol. This code would not be applied if the protocol allows flexibility in the order in which specific modules or interventions occur.

8. Integrating another approach into the intervention: The intervention of interest is used as the starting point, but aspects of different therapeutic approaches or interventions are also used (*e.g*., integrating an ‘empty chair’ exercise into a ‘CBT for Depression’ treatment protocol).

9. Integrating the intervention into another approach: Another intervention is used as the starting point, but elements of the intervention of interest are introduced (*e.g*., integrating motivational enhancement strategies into a weight loss intervention protocol).

10. Repeating elements: One or more modules, sessions, or activities that are normally prescribed or conducted once during a protocol are used more than once*.*

11. Loosening structure: Elements intended to structure intervention sessions do not occur as prescribed in the manual/protocol (*e.g*., the ‘check-in’ at the beginning of a group intervention is less formally structured; clinician does not follow an agenda that was established at the beginning of the session).

12. Departing from the intervention (‘drift’): The intervention is not used in a particular situation or the intervention is stopped, whether this stoppage was for part of a session or a decision to discontinue the intervention altogether (*e.g*., ‘this client was so upset that I just spent the rest of today’s session letting him talk about it instead of addressing his health behaviors’).

## Discussion

This study represents an effort to systematically characterize the types of modifications that are made to interventions when they are implemented in real world settings. On a high level, two of the major categories of coding mapped onto Castro and colleagues’ distinction between modifications of program content, and modification of the form of delivery (*e.g*., location of delivery, delivery person, or channel of delivery) [27]. In a sample of studies described in peer-reviewed articles, which represent a variety of interventions and contexts, we found that contextual modifications were occasionally reported, but that content modifications were reported much more frequently. Tailoring the intervention to address language, cultural differences, literacy, or situational constraints was the most commonly identified content modification, followed by the addition or removal of elements and changes to the length or pacing of the intervention.

Other modifications identified in our coding process, such as drift and loosening of structure, occurred relatively rarely within the articles that we reviewed. This low frequency in the current sample is not surprising, as such behaviors are unlikely to occur in a planned manner, and may be less likely to be emphasized when describing an evidence-based intervention in a peer-reviewed article. Furthermore, relatively few of the articles that were sampled employed the type of observation or stakeholder interviews through which such behaviors may be identified. While drift might also be considered a discontinuation of the intervention entirely or a lack of fidelity rather than a modification, it also seems important to capture it in a system designed to classify deviations from and modifications to a protocol in order to better measure its impact on outcomes of interest. For example, the impact of the option to occasionally or strategically drift on clinician or client satisfaction may be important to explore, in addition to the impact of drift on clinical effectiveness.

In contrast to the findings in Hill and colleagues’ study [20], most of the articles that we found in our search process described modifications that were made proactively in recognition of key differences between the implementation setting and the original intervention. In another report, we describe findings that emerged when we applied this framework to interview data from a sample of community-based mental health service providers who were trained in an EBP [53]. Several of the lower-frequency modifications identified in the current study were endorsed much more frequently in that study, suggesting that modifications made proactively may differ from those made once implementation is underway. Thus, at this stage of development, we determined that it is important to represent a more exhaustive set of possible modifications in the classification system.

As the discussion above indicates, some modifications may signify decreases in fidelity, while others may be consistent with the design of the intervention. The tension between modification and fidelity is a critical issue in implementation science [4,54,55]. Many recognize that modifications will occur throughout the course of an implementation effort, but the type and extent of modifications that can occur without compromising effectiveness or degrading fidelity to an unacceptable degree has not been sufficiently explored. In theory, it is possible to make some types of modifications without compromising effectiveness or removing the key elements of an intervention. However, for some interventions, the core elements have not yet been determined empirically, and very little is known about the impact of behaviors such as integrating other interventions or selectively implementing particular aspects of a treatment. Fidelity measures that emphasize competence or the spirit of an intervention over adherence may not adequately capture some potentially important types of modification, and those that emphasize adherence may not capture modifications such as tailoring. Thus, when observation or reliable self-report is possible, the use of a fidelity measure along with this modification framework can guide decisions regarding the extent to which a particular modification represents a departure from core elements of an intervention. Used alone or as a complement to fidelity measures, this measure may also be useful in determining whether particular elements can be removed, re-ordered, integrated or substituted without compromising effectiveness.

Despite the breadth of the coding system we developed, interrater agreement for the subset of independently-coded articles was quite high, reaching standards of ‘substantial agreement’ and ‘almost perfect’ agreement for the level and nature of modifications, respectively [52]. Within our research group, this level of reliability was achieved after a brief series of hour-long weekly coding meetings, suggesting that our coding scheme can be used to reliably classify modifications described in research articles without overly burdensome training.

We note several potential limitations to the study and framework. First, our search process was not intended to identify every article that described modifications to evidence-based interventions, particularly if adaptation or modification was not a major topic addressed in the article. Instead, we sought to identify articles describing modifications that occurred across a variety of different interventions and contexts and to achieve theoretical saturation. In the development of the coding system, we did in fact reach a point at which additional modifications were not identified, and the implementation experts who reviewed our coding system also did not identify any new concepts. Thus, it is unlikely that additional articles would have resulted in significant additions or changes to the system.

In our development of this framework, we made a number of decisions regarding codes and levels of coding that should be included. We considered including codes for planned vs. unplanned modifications, major vs. minor modifications (or degree of modification), codes for changes to the entire intervention vs. changes to specific components, and codes for reasons for modifications. We wished to minimize the number of levels of coding in order to allow the coding scheme to be used in quantitative analyses. Thus, we did not include the above constructs, or constructs such as dosage or intensity, which are frequently included in frameworks and measures for assessing fidelity [56]. Additionally, we intend the framework to be used for multiple types of data sources, including observation, interviews and descriptions, and we considered how easily some codes might be applied to information derived from each source. Some data sources, such as observations, might not allow coders to discern reasons for modification or make distinctions between planned and unplanned modifications, and thus we limited the framework to characterizations of modifications themselves rather than how or why they were made. However, sometimes, codes in the existing coding scheme implied additional information such as reasons for modifying. For example, the numerous findings regarding tailoring interventions for specific populations indicate that adaptations to address differences in culture, language or literacy were common. Aarons and colleagues offer a distinction of consumer-driven, provider-driven, and organization-driven adaptations that might be useful for researchers who wish to include additional information regarding how or why particular changes were made [35]. While major and minor modifications may be easier to distinguish by consulting the intervention’s manual, we also decided against including a code for this distinction. Some interventions have not empirically established which particular processes are critical, and we hope that this framework might ultimately allow an empirical exploration of which modifications should be considered major (*e.g*., having a significant impact on outcomes of interest) for specific interventions. Furthermore, our effort to develop an exhaustive set of codes meant that some of the types of modifications, or individuals who made the modifications, appeared at fairly low frequencies in our sample, and thus, their reliability and utility require further study. As it is applied to different interventions or sources of data, additional assessment of reliability and further refinement to the coding system may be warranted.

An additional limitation to the current study is that our ability to confidently rate modifications was impacted by the quality of the descriptions provided in the articles that we reviewed. At times, it was necessary to make some assumptions about how things were actually modified, or the level at which the modifications occurred. The level of detail available in records, clinical notes, or other qualitative data that may be utilized to investigate modifications may similarly impact future investigations. We attempted to address this limitation by making decision rules about the level of detail and clarity required to assign codes and by documenting these rules in detail in our coding manual. The level of rater agreement that we achieved suggests that our process was reasonably successful, despite occasional ambiguities in the descriptions. In future efforts to utilize this system, two strategies can minimize the likelihood that insufficient data are available to assign codes. Whenever possible, observation by raters knowledgeable about the intervention and its core components should be used to identify modifications. This may be especially important in differentiating minor modifications (which might be coded as ‘tailoring/tweaking/refining’) from more intensive modifications (which, for example, might be coded as ‘removing elements’); ultimately, making these distinctions requires a thorough knowledge of the intervention itself. When interviews are conducted in lieu of observation or in addition to review of existing records, we recommend asking very specific follow-up questions regarding modifications that are made. Familiarity with both the intervention and the coding system when interviewing can increase the likelihood that sufficient information is obtained to make an appropriate judgment. Despite these measures, interrater reliability may vary across different data sources, although additional work by our research group suggests that reliability remains high when the coding scheme is applied to interview data [53]. We are currently examining reliability when the coding scheme is used for observation using audio recordings of psychotherapy sessions as well, and we recommend that when using this framework, researchers assess reliability.

We believe that the framework that we present can be used flexibly depending on the goals of the research and the type of data collection that occurs. For example, researchers may wish only to code exclusively for content or context-level modifications if they are interested in determining the impact of specific types of modifications on health outcomes. Similarly, the code for the decision maker may not be necessary if researchers are studying modifications made by one particular group or evaluating adaptations that were pre-specified by a single decision-maker before implementation began. However, this code might be very informative if the researchers wish to understand the impact of the nature and process of modification on outcomes such as stakeholder engagement or fidelity to core program or intervention elements.

This coding system may be used to advance research that is designed with the goal of understanding the impact of changes made to interventions in particular contexts. Ultimately, such an understanding will require simultaneous use of this coding scheme and treatment outcome assessments, in order to help researchers and clinicians determine what specific types of modifications are most useful in increasing the effectiveness of interventions. Such an understanding will allow stakeholders to make more informed decisions about whether and how to modify the interventions when implementing them in contexts that differ from those in which they were originally developed and tested. Additionally, when used in the context of fidelity monitoring, this system can provide more useful information about what actually occurs when lower levels of adherence are identified, as well as the types of modifications that can occur within acceptable levels of fidelity. Baumann and colleagues suggested that there is a range of feasible fidelity, as well as a point of ‘dramatic mutation,’ at which the intervention is no longer recognizable or effective [8]. This system of characterizing modifications may be useful in determining these ranges and boundaries with greater specificity. By understanding the types of modifications that can be made while keeping the intervention out of the range of dramatic mutation, stakeholders may ultimately find it easier to adapt interventions as needed while attending to an intervention’s most critical components. Investigations of the impact of particular types of modifications on clinical outcomes can further inform efforts to implement evidence-based interventions while preserving desired levels of effectiveness. Finally, another potential area of investigation using this framework is on the impact of specific modifications on implementation outcomes such as adoption and sustainability. Additional knowledge about these critical issues in implementation science will yield important guidance for those wishing to advance the implementation of evidence-based programs and interventions.

## Competing interests

The authors declare that they have no competing interests.

## Authors’ contributions

SWS conceptualized the study, contributed to the data collection and coding, and was the predominant contributor to this article. CM assisted with data analyses, assessment of reliability, interpretation of results, and contributed to the text of the manuscript. KT and AC assisted with literature searches, codebook development, and coded articles in the study. All authors did critical reading and modification of drafts and approved the final manuscript.
